# Citizen Science in Air Pollution Monitoring: Methodological Approaches, Effectiveness, and Integration with Health Research

**DOI:** 10.1007/s40572-026-00559-1

**Published:** 2026-08-28

**Authors:** Hong Yang

**Affiliations:** Department of Geography and Environmental Science, University of Reading, Reading, RG6 6AB UK

**Keywords:** Citizen science, Air pollution monitoring, Low-cost sensors, Environmental health, Community engagement, Exposure assessment

## Abstract

**Purpose of review:**

This review critically examines the role of citizen science in air-pollution monitoring and its integration into environmental health research. It explores how participatory approaches, including low-cost sensors and community co-creation, can enhance spatial resolution, public engagement, and environmental justice, while assessing data quality, health relevance, and scalability.

**Recent findings:**

Recent studies document the expanding global deployment of citizen science air quality monitoring networks. Advances in calibration algorithms, wearable technologies, and participatory data-collection methods have strengthened capacity to assess real-time exposure. Several projects demonstrate the increasing technical sophistication and policy relevance of citizen-generated air quality data.

**Summary:**

Citizen science provides hyperlocal insights into air quality and exposure, strengthens risk communication, and contributes to health-impact assessment. Although challenges remain regarding data reliability, representativeness, and policy uptake, emerging approaches, including AI-assisted calibration and distributed sensor networks, offer promising pathways forward. An integrative framework can support sustained community engagement and evidence-informed action to improve air quality.

## Introduction

Air pollution is a critical global environmental and public health challenge, associated with approximately 6.7 million premature deaths worldwide each year [1]. Traditional regulatory air quality monitoring relies on a sparse network of expensive, stationary stations that provide highly accurate data but with limited spatial coverage and coarse resolution [2]. Many cities have only a handful of monitors, leading to an incomplete picture of localized pollution variability. To address these gaps, recent years have seen the rise of citizen science approaches in air quality monitoring, empowered by low-cost sensors and participatory methods. Citizen science in this context involves public volunteers collaborating with scientists to collect and analyze air pollution data, often using affordable, portable monitoring devices or simple sampling tools in their communities [3, 4]. These grassroots monitoring initiatives greatly increase the spatial and temporal density of air quality observations at a fraction of the cost of regulatory stations.

Citizen-driven air quality monitoring has rapidly expanded in the last decade, reflecting growing public interest and technological advances [2, 5–16] (Table 1). By democratizing environmental data collection, citizen science offers a bottom-up complement to traditional top-down monitoring, engaging local knowledge and concerns in the scientific process. This review paper examines the current landscape of citizen science approaches to air pollution, evaluating their methodologies, effectiveness, and limitations. This paper synthesize findings from studies across global projects, including the use of low-cost particulate matter (PM) and gas sensors, passive samplers, mobile monitoring, and co-created community campaigns. Key themes include data quality (accuracy, calibration, comparability with reference stations), spatial–temporal coverage, and the social dimensions of participation. This paper also assesses how citizen-generated air quality data contributes to understanding human exposure and health impacts, by linking community monitoring with exposure assessment, epidemiology, and risk communication. Building on these insights, this paper proposes a conceptual framework that connects citizen science monitoring to environmental health research and policy. Finally, this paper identifies research gaps and future directions, highlighting emerging technologies (e.g., AI for sensor calibration, wearable exposure trackers, sensor networks), ethical considerations, and implications for policy and environmental justice.

**Table 1:** Summary of selected citizen science air quality monitoring studies, including project location, pollutants and sensor types, methodology, data accuracy, main findings, and noted health or policy implications

Study (Location)	Pollutant & Sensor Type	Methodology & Scale	Data Accuracy/ Calibration	Main Findings	Health / Policy Implications	References
CurieuzeNeuzen Vlaanderen (Flanders, Belgium)	NO_2_ passive diffusion tubes (Palmes tubes)	20,000 citizens each deployed a tube for 1 month. Data collected via mail-back, mapped by researchers.	Tubes calibrated against reference NO_2_ monitors before/after deployment; achieved high data quality (error ± 10–20%). Rigorous QA/QC led to high correlation (*R* ~ 0.8–0.9) with reference data after calibration.	Generated an unprecedented high-resolution NO_2_ concentration map for the entire region. Revealed fine-grained pollution patterns: e.g. higher NO_2_ on average along busy roads vs. nearby streets. Identified numerous local hotspots previously unknown. Data used to validate and improve a regional air quality model.	Brought public and media attention to traffic pollution; informed policy by highlighting streets exceeding EU limits. Enabled estimation of health impacts – e.g. suggested that reducing NO_2_ in hotspots could significantly cut asthma incidence. Empowered citizens and authorities with better evidence; follow-up measures included low-emission zones in affected areas.	[6, 7]
xAire School Monitoring (Barcelona, Spain)	NO_2_ passive samplers (Gradko tubes)	Co-created campaign at 18 primary schools (1,650 participants). Placed 725 samplers around schools for 1–2 week intervals, capturing street-level NO_2_. Community workshops held on data and pollution sources.	Samplers analyzed in lab; collocated comparison at reference sites (R² ≈0.8). Land-use regression (LUR) model applied to calibrated data to produce 100 × 100 m resolution NO_2_ map. Uncertainty in LUR estimates reduced by dense sampling.	Found very high NO_2_ near some schools, with clear variation by street traffic volume. Provided high spatial exposure data for children. A substantial proportion of school-related sites were found to exceed the WHO 2021 Global Air Quality Guideline for annual mean NO₂ of 10 µg/m³, and many also surpassed the former 40 µg/m³ guideline.	Health impact assessment conducted using the citizen data: estimated > 1,000 annual cases of childhood asthma could be prevented by lowering NO_2_ to background levels. Results galvanized policy action: city accelerated creation of “school superblocks” (traffic restrictions) around high-NO_2_ schools. Demonstrated how citizen data can feed directly into public health analysis and local policy.	[8]
Imperial County Community Air Network (CA, USA)	PM_2.5_ and PM_10_ – low-cost optical sensors (Beta Attenuation Monitors and Dylos units)	Community-designed network in rural community (40 sensors at schools, clinics, etc.) streaming real-time data. Residents involved in siting and maintenance; data on open platform (IVAN Air) for public and agencies.	Field validation study published: calibrated sensor readings to gravimetric reference (R² ~0.7–0.8 for 24-hr PM_2.5_). Co-location indicated sensors slightly over-read at high concentrations; project applied correction factors. Quality assurance with periodic swaps and reference checks.	Identified strong spatial patterns of particulate pollution (e.g. highest PM near desert dust sources and Salton Sea shoreline). Data revealed 10× higher PM spikes during windblown dust events than regional monitors recorded, explaining community health complaints. Real-time data used by schools to implement exposure-reduction measures on high-pollution days (indoor recess, etc.).	Directly addressed an environmental justice gap – provided actionable information in a region with high asthma rates and few prior monitors. Data has been used by local health department and clinicians for asthma intervention programs. Informed state policy: project became a model for California’s AB 617 community air networks. Empowered residents: community members gained scientific agency and successfully lobbied for stricter enforcement on agricultural dust controls using project data as evidence.	[9–12]
Personal Exposure Monitoring Study (United Kingdom)	PM_2.5_ – portable nephelometer sensor carried by individuals	30 adult volunteers carried wearable PM monitors for 24-hour periods, keeping diaries of locations (indoor/home, outdoor, transit). Also placed static indoor and outdoor reference monitors at select homes for comparison.	Research-grade portable monitors (factory-calibrated, checked against gravimetric PM filters). Accuracy ~ ± 15%-20% for 1-min data. Time-resolved personal exposures compared with nearest fixed background monitor. Validation: personal monitor data closely tracked reference instruments when side-by-side (*R* ~ 0.80–0.95).	Demonstrated that personal PM_2.5_ exposures can differ greatly from ambient levels. Commute microenvironment had 2–4× higher PM than home background for many participants; indoor exposures during cooking were significant. Found poor correlation between an individual’s exposure and city monitor data at the same time, underscoring importance of personal monitoring for exposure assessment.	While not a health outcome study, it highlighted potential misclassification in epidemiology if relying only on central monitors. Underscored need to incorporate microenvironment exposure in health risk analysis. Results were used in risk communication: participants received personalized advice (e.g., using kitchen ventilation, taking quieter walking routes) to reduce their pollution dose. The study also validated methodologies for larger-scale use of wearables in citizen health studies.	[13, 14]
SoCal Disadvantaged Communities Study (Southern California, USA)	PM_2.5_ – PurpleAir PA-II low-cost sensors (optical laser particle counter)	22 households in 8 disadvantaged (low-income, minority) communities installed outdoor PA-II sensors, monitored continuously for 6 months. Community members were trained and participated in data interpretation workshops.	Used US EPA’s correction equation for PurpleAir to calibrate readings to equivalent federal standard. Validation: 2 sensors co-located with regulatory monitors showed improved accuracy (R² >0.8) after correction vs. R² ~0.5 raw. Uncertainty ~ ± 2–3 µg/m³. Data aggregated to hourly and daily averages for analysis.	Found that communities with higher proportions of Hispanic/African-American residents and poverty had significantly higher PM_2.5_ concentrations than more affluent nearby areas. Clear seasonal trend: winter PM_2.5_ (~ 25.8 µg/m³) was ~ 2× summer levels, likely from residential heating and stagnant weather. Also identified distinct daily patterns (e.g. overnight PM buildup in inland valleys). Mapped local “hotspots” near freeways and warehouse clusters, aligning with known diesel emission sources.	Provided empirical evidence of environmental inequality in air pollution exposure. Results are informing local environmental justice campaigns and urban planning—e.g. supporting calls for buffer zones around warehouses and traffic in these communities. From a health perspective, the higher sustained PM_2.5_ levels in environmental justice areas indicate greater risk of pollution-related diseases, bolstering the case for targeted public health interventions. The project also demonstrated feasibility of engaging residents in sustained monitoring, building community capacity to understand and act on air quality issues.	[15, 16]

### Citizen Science Methods for Air Quality Monitoring

Modern citizen science air projects predominantly utilize low-cost air pollution sensors to measure common pollutants such as PM_2.5_, PM_10_, NO_2_, O_3_, and others. These sensors include optical particle counters for PM and electrochemical cells for gaseous pollutants, often integrated into small, battery-powered devices or sensor “pods”. They are inexpensive (typically $50–$300 per unit) and compact, making it feasible to deploy dozens or thousands of monitors by volunteers. Fixed sensors are installed outside homes, schools, or at community sites, creating dense networks that capture fine-grained spatial patterns [17]. For example, the global Sensor.Community network has empowered citizens to install tens of thousands of PM sensors, yielding open-data maps of urban air quality with far greater coverage than official stations [18]. Such expansive networks can reveal pollution hotspots even within neighborhoods, information that was previously unattainable. Mobile monitoring is another approach: citizens carry portable sensors on bikes, cars, or on-person to map pollution along their daily routes. Notably, projects like “Snifferbike” in the Netherlands equipped cyclists with PM_2.5_ sensors to crowd sample street-level air quality, demonstrating how mobile sensors capture micro-scale variations (e.g., higher PM on busy roads) and personal exposure during commutes [19]. Wearable devices and smartphone-based sensors now allow individuals to log their real-time exposure as they move through various microenvironments [20]. These innovations support the “personalization” of air quality data, enabling people to measure their own exposure based on their activities and locations.

Not all citizen science relies on electronic sensors; passive sampling techniques are also widely used due to their simplicity and low cost. Passive diffusion tubes for NO_2_ or VOCs, for instance, can be distributed to hundreds of volunteers to deploy outside their homes or workplaces for a period (typically 4 weeks) [21]. They require no power and are relatively easy to handle, making them ideal for large-scale campaigns. The trade-off is that passive samplers only provide time-averaged concentrations over the exposure period and must be sent to a lab for analysis, so they lack real-time feedback [22]. Nevertheless, they have enabled extremely large citizen science projects. A landmark example is CurieuzeNeuzen Vlaanderen in Belgium, where 20,000 citizens deployed NO_2_ diffusion tubes across Flanders, yielding an unprecedented high-resolution pollution map [23]. This participatory campaign successfully covered urban and rural locations, identified traffic pollution hotspots, and provided data to validate and improve government air quality models. Similarly, the xAire project in Barcelona engaged parents and students at 18 schools to install passive NO_2_ samplers around schoolyards, generating granular data on children’s exposure that informed a health impact assessment [8]. Passive sampling campaigns exemplify “community sampling” approaches, wherein residents themselves become field data collectors on a mass scale. They illustrate how simple tools, when scaled through citizen participation, can produce policy-relevant scientific insights.

Citizen science air monitoring projects vary in their degree of public involvement, ranging from contributory models (scientists lead design; citizens collect data) to co-created models (citizens and scientists collaboratively design and execute the project). Many initiatives start as grassroots efforts in pollution-affected communities, often supported by NGOs or academia. For example, in Imperial County, California – a rural, underserved region with high particulate pollution – community members partnered with researchers to design a monitoring network that addressed local concerns [9, 12]. Citizens helped select 40 sensor locations (e.g., schools, clinics) and were trained to maintain devices, effectively becoming co-researchers. This co-creation and capacity building ensured the project reflected community priorities (like identifying farm dust and cross-border pollution sources) and built local technical skills. Other projects use a “citizen observatory” model, for example, Kids Action Thru Science (KATS) programme in Newcastle, the UK, providing online platforms and Apps where volunteers can view data, flag anomalies, or even perform basic analyses [24]. Whether contributory or co-created, successful projects establish feedback loops with participants, e.g., sharing results in accessible formats (maps, apps, workshops) and acknowledging contributors. This keeps volunteers motivated and enhances scientific literacy. Indeed, involving citizens in data analysis and discussion (not only data collection) can empower communities to interpret results and advocate for changes, as seen in Barcelona’s xAire campaign where public engagement translated data into collective action on traffic air pollution [8].

### Data Quality, Calibration and Validation

A common critique of low-cost sensors in citizen science is data quality. Ensuring scientific rigor requires careful calibration, validation, and data handling protocols in citizen projects. Many low-cost sensors have inherent measurement biases and higher uncertainty due to factors like sensor drift, cross-sensitivity (e.g., gas sensors responding to temperature or humidity), and manufacturing variability [25]. Without correction, raw data from citizen sensors can deviate significantly from reference-grade instruments. Recognizing this, researchers and agencies have invested in calibration frameworks for citizen sensor networks. A prominent approach is co-location calibration: deploying the low-cost sensors side-by-side with an official monitor for a period to derive correction factors or regression equations [26]. For example, the U.S. EPA and collaborators tested the popular PurpleAir PM_2.5_ sensors at over 70 regulatory sites and developed a nationwide correction equation to align PurpleAir readings with Federal Equivalent Method (FEM) monitor data [27]. Applying such calibrations greatly improves the accuracy of citizen sensors, making their data comparable to the public regulatory network. In fact, after correction, PurpleAir sensors achieved up to ~ 97% correlation with government monitors in some evaluations [28], highlighting that while uncalibrated low-cost sensors often have biases, they can be reliably used when properly calibrated and quality-controlled.

Beyond one-time factory or co-location calibration, dynamic calibration and machine learning techniques are emerging to continually adjust sensor data. For instance, field calibration models can incorporate environmental conditions (e.g., temperature and humidity) to correct drifting sensor output in real-time [29]. Recent studies demonstrate adaptive algorithms that improve low-cost sensor performance under varying humidity or pollution mixtures [30]. This is crucial for particulate sensors. Since optical PM readings can be skewed by humidity (water on particles) – condition-specific calibration (or humidity compensation formulas) are needed for accuracy [31]. Similarly, electrochemical gas sensors benefit from frequent re-calibration to account for baseline drift and interference from other gases [32]. Some citizen science projects schedule regular collocation of a subset of sensors at reference stations and update calibration curves accordingly (often termed “network calibration”) [33]. Others employ statistical data fusion, combining citizen sensor data with modelled or official data to bias-correct the observations [34].

Quality assurance in citizen science also involves data validation and filtering. Because volunteer networks can generate vast streams of data, automated systems are used to flag outliers or sensor malfunctions. For example, the OpenSense network in Switzerland applied algorithms to detect sudden jumps or flatlining in sensor readings indicative of faults, automatically excluding bad data from public feeds [35]. Furthermore, comparisons with reference monitors are periodically conducted to quantify accuracy. Field studies often report metrics like correlation (R²) with reference, or mean error, for example, low-cost NO_2_ sensor pods achieving R² ~0.7–0.8 against an official station after algorithm updates [36]. Passive samplers used by citizens in the CurieuzeNeuzen Vlaanderen project are typically analyzed in the same labs as regulatory filters, and their precision has been often within ~ 10–20% of reference measurements for NO_2_, under good handling [37].

Moreover, sensor drift over months requires recalibration or replacement [38], a practical challenge for volunteer-based projects. Many citizen science initiatives have addressed this by rotating sensors back to labs for post-deployment testing or by providing guidance to participants on periodic sensor check-ups. For example, the Imperial County community network performed field validation of their PM sensors for over a year and published performance results to ensure transparency [9].

The bottom line is that data quality practices are now a central component of citizen science air monitoring, involving multidisciplinary efforts (engineering, statistics, and community training) to make sure the data generated are trustworthy and usable.

### Comparing Citizen Air Quality Measurement with Regulatory Monitoring

Citizen science and regulatory monitoring should be viewed as complementary. Traditional monitors are highly accurate and produce long-term datasets needed for trends and compliance, but they are few and often located to capture area-wide pollution, not neighborhood spikes. Citizen networks, by contrast, offer hyperlocal insights and dense coverage but with higher noise [39]. A clear advantage of citizen sensor approaches is spatial granularity: they can uncover strong pollution gradients over short distances (e.g., between a busy roadway and a nearby park) that a city’s single central monitor cannot resolve [40]. They also enable high-frequency temporal data in more locations (e.g., some low-cost devices report every minute) which can capture transient pollution events (like fireworks, street-level rush hour peaks, or industrial upsets) often missed by averaging official data [34]. In Lisbon, Portugal, a new urban sensor network of 264 low-cost nodes provides real-time maps of air pollution and noise on an open-data platform, vastly expanding the city’s environmental monitoring footprint [17].

**Fig. 1:**
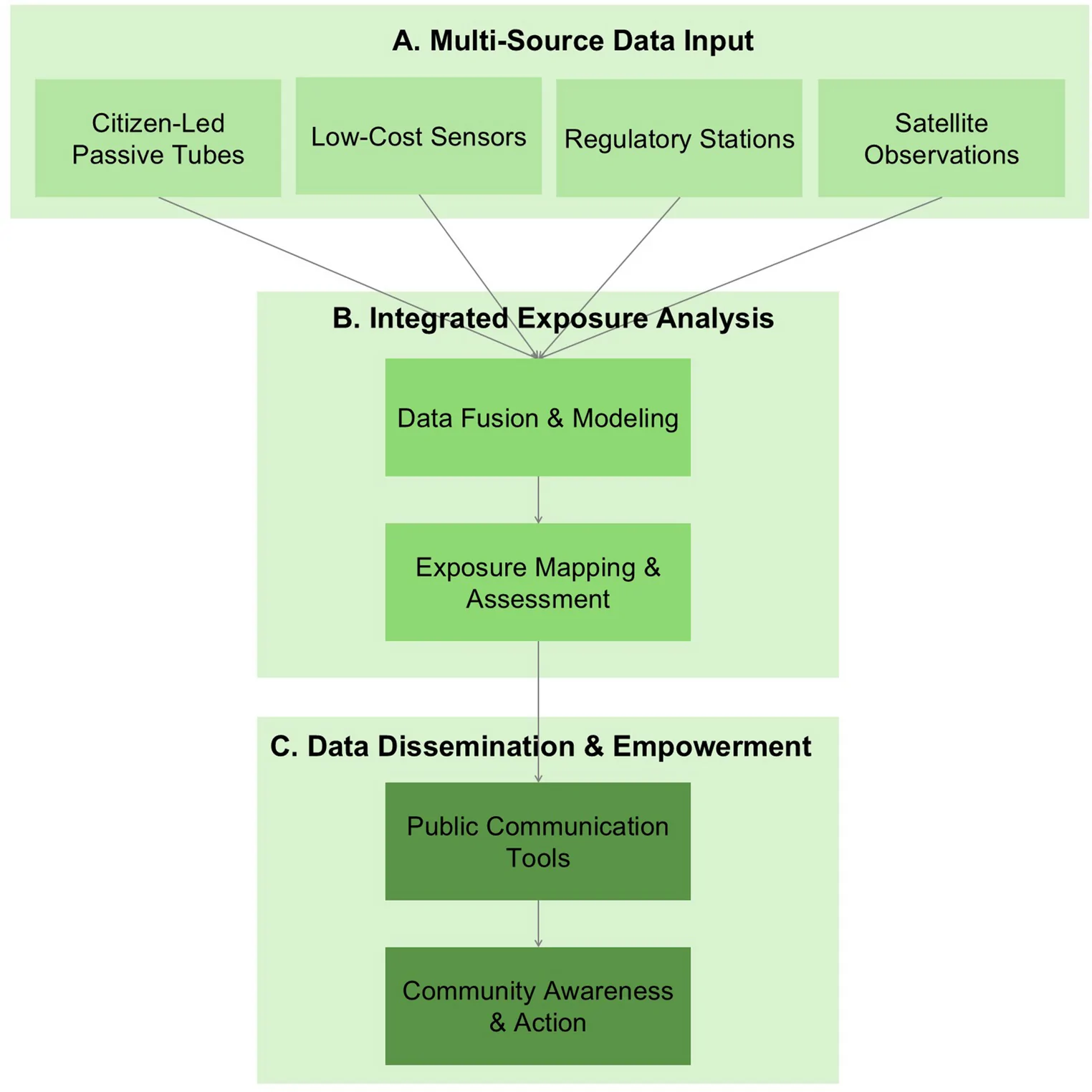
Conceptual framework for integrating citizen science and multi-source air quality data in exposure assessment and community empowerment. (**A**) Air pollution data are derived from complementary sources, including citizen-led passive samplers, low-cost sensors, regulatory monitoring stations, and satellite observations. (**B**) These heterogeneous datasets are combined through data fusion and modelling to produce spatially resolved exposure maps and assessments. (**C**) Results are disseminated via public communication tools to enhance community awareness, support informed action, and strengthen evidence-based engagement in air quality governance

On the other hand, regulatory stations undergo strict maintenance, audits, and calibration, ensuring data of legal defensibility, but volunteer networks typically cannot guarantee. Citizen sensed data often faces skepticism regarding accuracy and standardization. However, even though low-cost sensors do not achieve the same level of performance as high-cost regulatory-grade instruments, they can nevertheless generate useful and detailed air quality data that inform policy decision-making. In practice, environmental agencies are increasingly embracing citizen data to fill gaps [41]. The U.S. European Environment Agency (EPA) has explored using citizen passive sampler results to complement modelled maps. U.S. EPA’s AirNow Fire and Smoke Map integrates corrected crowdsourced sensor readings alongside official monitors, so the public sees a more comprehensive picture [41]. (Fig. 1) conceptualises this integrative approach. Multiple data sources—including passive diffusion tubes, citizen-operated low-cost sensors, in-situ regulatory monitoring stations, and satellite observations—feed into integrated analyses. When translated effectively, these multi-source data streams empower individuals and communities with actionable knowledge, bridging the gap between static network averages and the personal exposures people actually experience.

Rather than viewing one as replacing the other, a hybrid approach to urban air quality management should be adopted: citizen monitoring networks can be used to identify relative differences and hotspots, followed by the deployment of reference instruments or targeted controls in those locations [42]. Citizen data can also be an early screening tool, highlighting areas of concern that merit official investigation (for instance, a neighborhood’s sensors repeatedly showing high nighttime VOCs could trigger authorities to inspect nearby industry). Conversely, citizen efforts benefit from regulators’ support in training, data validation, and technical guidance (many successful projects, like Imperial County’s, had agency or academic partners providing this support [9]). Ultimately, an inclusive monitoring paradigm is emerging, where government, scientists, and citizens collaborate to produce a more complete and democratically accessible understanding of air quality.

### Strengths and Benefits of Citizen Science Air Quality Measurement

Compared with traditional approaches, citizen science in air pollution research offers several compelling strengths.

The first strength is hyper-local data and novel insights. By vastly increasing the number of observation points, citizen networks capture spatial variations at the street and neighborhood level that were previously invisible in official data [43]. This hyper-local granularity can identify pollution “hotspots” (e.g., a specific traffic corridor or a poorly ventilated street canyon) and microscale patterns (e.g., higher PM_2.5_ on routes frequented by heavy-duty trucks) that enable targeted mitigation [34]. For example, the CurieuzeNeuzen project in Flanders, Belgium, revealed substantial NO_2_ differences not just between cities and rural areas, but within cities block-by-block, information used to fine-tune traffic abatement strategies [44]. These insights into local exposure differences are especially relevant for environmental justice, as they can highlight disadvantaged communities bearing disproportionate pollution burdens that averages might mask [15].

The second strength is community engagement and empowerment. Citizen science actively involves the public in environmental monitoring, demystifying science and empowering communities. Participants gain knowledge about air pollution and its sources, learning by doing. Many report increased environmental awareness and advocacy as a result of taking part [8]. Citizen monitoring has motivated communities to push for policy changes, for instance, parents engaged in the xAire school monitoring campaign in Barcelona became vocal champions for cleaner air around schools, contributing to the city’s decision to implement “school streets” (traffic restrictions near schools) [8]. In low-income communities with historical mistrust of authorities, community-owned data can be a powerful tool. Residents of Imperial County (California, US) took pride in “their” network’s data and used it in meetings with regulators to demand action on dust control [9]. This empowerment dimension, giving people the means to scientifically document their lived environment, is a unique social benefit of citizen science that goes beyond the data itself.

The third strength is bridging knowledge gaps (Science–Society–Policy). Citizen science creates a more inclusive interface between scientists, policymakers, and the public. It serves as a catalyst for dialogue, and citizens become stakeholders in research rather than passive subjects [45]. This can improve trust in scientific findings and environmental policies, and the collaborative monitoring efforts are bridging gaps between communities and scientists by co-producing knowledge in an inclusive way [46]. Such collaborations can also inject local knowledge into research (for example, residents might point out odor events or local sources that automated analysis would miss) and ensure research questions align with community concerns. Ultimately, this can strengthen the science–policy–society interface: data generated has greater legitimacy in the eyes of the public, and policies informed by such data may enjoy greater public support [47]. Citizen science can improve decision-making by broadening the evidence base and stakeholder input [45]. For instance, urban plans that incorporate citizen air data may better address real community exposure patterns, making interventions more effective and fairer.

The fourth strength is rapid and cost-effective expansion of monitoring. Low-cost sensors and volunteer participation enable coverage in places where building new regulatory stations would be impractical due to cost or logistics (e.g., rural villages). Citizen campaigns can be deployed relatively quickly and flexibly. For example, when wildfire smoke severely impacted regions with sparse monitoring, volunteer sensor networks (e.g., PurpleAir) filled the information void in real time [41]. Similarly, in parts of the Global South, citizen science is providing baseline air quality data in cities that lacked monitoring altogether [48]. The Internet of Things (IoT) nature of many citizen sensors (with cloud-connected data) means results can be aggregated and visualized almost instantly, which is invaluable for public awareness and timely responses (e.g. individuals checking a community sensor map to decide if it’s safe to exercise outdoors) [49]. This agility and low cost make citizen approaches attractive for addressing emerging air pollution challenges or conducting exploratory measurements before investing in permanent infrastructure [50].

The fifth strength is environmental justice and inclusivity. Because citizen science invites participation from the public, it can uplift voices of communities that are often underrepresented in scientific data. In environmental justice contexts, neighborhoods historically overburdened by pollution but underserved by monitoring, citizen projects allow residents to document their conditions and gain evidence to advocate for change [51]. A 2022 study in Southern California, for instance, engaged residents in disadvantaged communities to host sensors and confirmed that their neighborhoods experienced higher PM_2.5_ levels than wealthier areas, information that can guide targeted emission controls and urban planning for equity [15]. Citizen data has also been used in health outreach (e.g., teaching asthmatic families how to use low-cost monitors to identify and avoid pollution spikes) thereby directly benefiting vulnerable groups [52].

In essence, the strengths of citizen science air monitoring lie not only in the rich data produced (high-resolution, local, timely) but also in the process of engaging and educating the public, which yields social capital and community-driven solutions. These strengths make citizen science a powerful complement to traditional monitoring and an important component of modern environmental health research and governance.

### Challenges of Citizen Science Air Quality Measurement

Despite its promise, citizen science approaches to air quality come with some limitations and challenges that need to be acknowledged.

The first challenge is data accuracy and reliability issues. Low-cost sensors generally have higher measurement error, drift, and inter-unit variability compared to reference instruments [39]. They can be sensitive to environmental factors (temperature, humidity) and require frequent calibration [53]. Without proper calibration, citizen sensor data may be biased or inconsistent (e.g., optical PM sensors overestimating under high humidity, or electrochemical NO_2_ sensors drifting over time) [32, 54]. Sensor drift means data quality can degrade if not checked periodically [39]. A field evaluation of a citizen PM network over three years found that while precision remained acceptable, some sensor units gradually lost sensitivity [55]. Additionally, low-cost devices can suffer from downtime due to power or connectivity issues, leading to data gaps [56]. All these factors necessitate robust Quality Assurance/Quality Control (QA/QC) protocols (often beyond the capacity of volunteer operators alone).

The second challenge is limited pollutant scope and sensitivity. Most citizen science monitoring has focused on a few pollutants (notably PM_2.5_ and NO_2_) for which affordable sensors are available. Other harmful pollutants, such as ultrafine particles (PM_1_ and even PM_0.1_), SO_2_, and air toxics (e.g., benzene), are generally beyond the scope of citizen devices due to cost or complexity. Even for pollutants that are measured, low-cost sensors may have higher detection limits, making them less useful in areas with relatively clean air or for pollutants that are usually low [57]. For example, measuring compliance with strict NO_2_ standards (e.g., 10 ppb annual limit) is challenging with a low cost electrochemical sensor that has noise on the order of a few ppb [58]. Temporal resolution constraints also arise. Passive diffusion tube samplers integrate over weeks and cannot capture short pollution spikes [59], while some real-time sensors might average to 1-hour data to reduce noise, missing peak 5-min values relevant for acute exposure [60]. These limitations mean citizen networks might not detect certain issues (e.g., short-lived toxic plumes or compliance edge cases) that specialized monitoring would.

The third challenge is data management and analysis challenges. Citizen science campaigns can generate huge volumes of data, tens of millions of observations, which pose handling and analysis challenges. Cleaning noisy data, storing and sharing datasets, and performing advanced spatial modeling (e.g., land-use regression on citizen data) require expertise and computing resources that community groups may lack [61]. The heterogeneity of data (different sensor types, varying calibration status, etc.) complicates aggregation. There are also issues of standardization. Each project might use its own data formats, making it hard to combine results or compare across studies. Without careful management, there is a risk of information overload or misinterpretation of citizen science data [62].

The fourth challenge is sustainability and scalability. Many citizen monitoring efforts begin as short-term projects or research pilots. Keeping them running in the long term is difficult once initial funding or enthusiasm wanes. Sensors need maintenance (cleaning, recalibration, replacement every couple of years) [34], and sustaining a volunteer network requires ongoing engagement and support. Some projects have observed drop-off in participation over time or devices going offline as citizens move or lose interest [63]. Scaling up from a pilot to a city-wide network also faces logistical hurdles: coordinating hundreds of people, ensuring uniform protocols, and possibly dealing with bureaucratic permissions (for sensor placements) can be daunting. In some cases, the grassroots nature means networks remain fragmented, lots of small siloed projects rather than one coordinated system [64]. There is a need for institutional frameworks to support and integrate these efforts (e.g., national data platforms or community-of-practice networks) [64]. Without that, many citizen science projects might not achieve their full potential impact or might dissolve after producing a one-time dataset.

In highlighting these challenges, it’s clear that citizen science is not a panacea for air pollution monitoring. Data produced must be treated with caution, and results cross-checked with other evidence when possible. However, with thoughtful design, such as community partnerships to improve participation diversity, and scientist involvement to ensure robust calibration and analysis, many limitations can be mitigated. Awareness of these challenges is crucial so that stakeholders (from health researchers to policymakers) neither over-interpret citizen science data nor dismiss it outright. Instead, understanding the uncertainties and biases allows for responsible use of citizen science findings as part of a weight-of-evidence approach in air quality management [65].

### Contributions to Exposure Assessment and Health Research

One of the most significant promises of citizen-collected air quality data is its application to exposure science and public health research [61]. By providing high-resolution pollution measurements in communities and even at the personal level, citizen science can enhance our understanding of human exposures and associated health effects in ways that traditional monitoring alone could not easily achieve [66].

Public health studies, such as epidemiological analyses of air pollution impacts, rely on estimating individuals’ pollutant exposures. Conventional exposure assessment often uses data from a few central monitors or broad models, which may misrepresent personal exposure (since people move between microenvironments and indoor/outdoor settings) [67]. Citizen science has begun to fill this gap by generating data at the scales relevant to actual human experience. For example, personal monitoring studies with wearable sensors have directly measured the PM_2.5_ that people inhale during daily activities [68]. Volunteers in the UK were equipped with portable PM sensors, finding that personal exposures could vary dramatically even within the same city and that commuting and indoor cooking were major determinants of air pollution peaks [69, 70]. Such findings underscore that an individual’s true exposure can differ from ambient concentrations measured at a distant fixed site. By integrating citizen-gathered microenvironment data (home, transit, work) with time-activity diaries, researchers can build more accurate exposure models that account for population behavior patterns [71]. This participatory exposure assessment approach yields richer data for epidemiology, potentially reducing exposure misclassification and strengthening associations between pollution and health outcomes.

Moreover, citizen networks provide spatially resolved exposure fields that can be used in health studies. Dense networks in urban areas allow the development of high-resolution pollution maps (e.g., via land-use regression (LUR) models or interpolation of sensor data) [72]. These maps can then be linked with health datasets at the community or even individual address level. In Barcelona, the xAire citizen data enabled researchers to update LUR models of NO_2_ with unprecedented fine scale, which were then applied to estimate children’s exposure at each school and to conduct a health impact assessment for childhood asthma [8]. The health impact assessments (HIA) suggested that lowering NO_2_ concentrations (to meet WHO guidelines) could prevent over 1,000 new asthma cases annually in the city’s children [8]. Similarly, in Flanders, Belgium, the CurieuzeNeuzen NO_2_ map was used to refine exposure estimates for epidemiological studies on cardiovascular health, providing more confidence in exposure rankings of different towns than previously possible with just a few monitors [7].

Citizen science has proven especially valuable in community-led health inquiries, often in environmental justice contexts. When residents suspect a link between local pollution sources and health problems (e.g., asthma prevalence, nuisance symptoms, or unusual cancer clusters, ), there is often insufficient official data to investigate. By collecting their own air quality measurements, communities can obtain evidence to evaluate these concerns. One example comes from the industrial fence-line communities in Denver, Colorado, where residents used low-cost sensors to log odor events and PM spikes near refinery operations [73]. The collected data helped correlate specific emissions with times when residents experienced health symptoms (e.g., headaches or nausea), building a case for regulatory action on odors. In Imperial County, California, the community PM network was initiated in part because of alarmingly high asthma emergency room visit rates; with real-time air data now available, local health agencies have been able to issue school warnings on bad days and study the impact of dust episodes on hospital visits [9]. Thus, citizen monitoring not only provides exposure data but becomes a tool for community epidemiology, engaging citizens in collecting both environmental data and sometimes health data (through surveys or symptom tracking) to jointly explore exposure-health links [74].

Additionally, citizen data contributes to identifying susceptible subgroups and microenvironments. For instance, by placing sensors at schools and senior centers [75, 76], citizen projects can highlight the pollution levels in places frequented by vulnerable groups (children, the elderly) and during critical time windows (e.g., school drop-off). This has direct relevance to exposure assessment for those subpopulations in health research. If a study is examining effects of air pollution on children’s lung development, having data from the immediate surroundings of schools, via citizen samplers, can improve each child’s exposure estimate versus relying on urban background levels. Wearable sensor projects have also revealed that individuals’ personal exposure can at times exceed ambient levels [77]. For example, a person cooking indoors without proper ventilation may experience higher PM_2.5_ than recorded outside [78]. While this example concerns indoor environments, it underscores that exposure is fundamentally personal. Citizen science enables the measurement of individualised exposure profiles, which can, in turn, inform targeted health advisories, for example, by educating people with asthma that indoor sources can contribute substantially to total exposure [79], rather than exposure being driven solely by outdoor pollution.

Another important contribution of citizen science to health outcomes is through risk communication and prompting behavior change [80]. When people collect and see their own air quality data, the risk from air pollution becomes more tangible and understandable [81], potentially motivating protective actions. For example, an individual who sets up a sensor at their home may learn that pollution peaks at certain times (e.g., during morning traffic) and decides to shift their exercise routine or commute time accordingly. Community groups often use citizen data to educate neighbors, holding meetings to explain the readings and advise on actions like keeping windows closed during high pollution periods or creating clean-air rooms [82]. The availability of hyperlocal data can also support vulnerable individuals in exposure self-management; for instance, asthmatics can consult a neighborhood sensor network (or carry a personal monitor) to know when to use inhalers or avoid outdoor exertion [83].

Importantly, citizen science projects often include an element of co-learning and dissemination. Participants become “ambassadors” who share pollution information with their peers, effectively broadening the reach of risk communication beyond what authorities alone can do [82]. The data visualization tools that accompany many citizen projects (interactive maps, smartphone apps, SMS alerts) are designed with the public in mind, translating complex pollution metrics into understandable formats (e.g., color-coded indices or simple messages). For instance, the AirNow smoke map integration of citizen sensors provides a user-friendly interface that millions have accessed to guide their activities during wildfire events [84]. When people are engaged with data, as citizen science fosters, they are more likely to trust the information and follow health advice (since they had a hand in producing the data, it’s more convincing than an impersonal alert). This can lead to measurable behavior changes, such as parents driving less or altering routes to reduce children’s exposure after seeing citizen monitoring results around schools [85].

At a broader policy level, citizen sourced data is increasingly being used to inform HIA and burden of disease studies by providing fine-grained exposure inputs [86]. The example of xAire in Barcelona yielded an updated asthma HIA [8]. In the research domain, epidemiologists are incorporating citizen data to refine exposure matrices in cohort studies. In the UK, citizen NO_2_ monitoring in London boroughs has been used in health equity analyses [42]. The citizen data gave the neighborhood-level resolution needed for such analysis. It allows researchers to ask new questions. For instance, how do intra-urban pollution disparities contribute to health outcome disparities, with data to back up the analysis [87]? 

Overall, citizen-generated air quality data is proving valuable for improving exposure estimates, engaging communities in health-related science, and directly informing both individual and policy-level decisions to mitigate health risks. The convergence of citizen science and environmental health research is an exciting development that stands to enrich our understanding of pollution exposure and empower communities to protect their health.

### Conceptual Framework Linking Citizen Science to Environmental Health

**Fig. 2:**
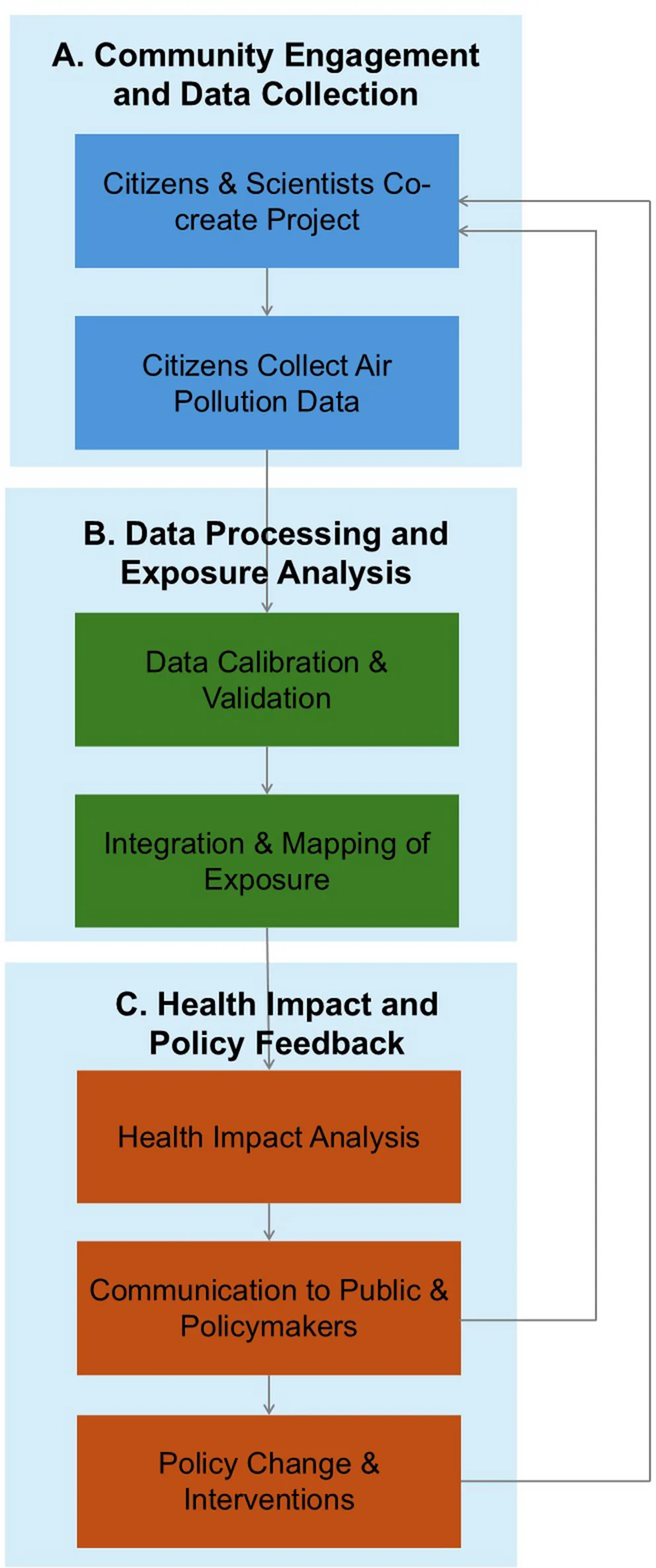
Conceptual framework for citizen science in air quality and health research. (**A**) Citizens and scientists co-design monitoring initiatives and collect air pollution data in response to community concerns. (**B**) Data are calibrated, validated, and integrated to generate exposure metrics (e.g., maps and datasets). (**C**) These outputs inform health impact assessments and are communicated to the public and policymakers, supporting policy interventions. Feedback loops highlight the iterative nature of the process, whereby communication and policy actions stimulate renewed community engagement and continued monitoring to evaluate outcomes

Drawing together the insights from this review, a conceptual framework (Fig. 2) is proposed to illustrates how citizen science air monitoring can be integrated into environmental health research and policy processes. This framework traces the path from community engagement and data collection through exposure assessment, health impact analysis, and policy action, with feedback loops that sustain the cycle. The framework underscores that citizen science is not a one-off data gathering exercise, but rather part of an iterative system that can influence and inform public health decision-making while empowering communities [88]. Key components include:

- Community Engagement & Co-Creation: The process begins with citizens and scientists identifying local air quality issues and co-designing a monitoring strategy (e.g., choosing sensor locations or study goals based on community concerns). This engagement ensures the research is relevant and that the community is invested in outcomes.
- Data Collection (Citizen Monitoring): Citizens collect data using low-cost sensors, passive samplers, mobile devices, and others. The raw data might be collected by individuals but often flows into a common platform. At this stage, training and support are crucial to maintain data quality (e.g., proper sampler deployment, device upkeep).
- Calibration & Validation (QA/QC): Before use in analysis, citizen data undergo calibration (via reference comparisons or algorithms) and validation to filter errors. This step, often supported by experts or agencies, translates raw volunteer data into scientifically robust datasets. It is the quality “gatekeeper” that allows the evidence to be credible.
- Data Integration & Analysis: The calibrated citizen data can then be integrated with other data sources (regulatory monitors, satellite data, and models) and subjected to analysis. Spatial interpolation, mapping, or modeling (like land-use regression) converts point measurements into exposure fields. Temporal analysis may identify pollution episodes or trends. Citizens may also participate in interpreting maps and results, adding local context.
- Exposure Assessment: Using the analyzed data, researchers or health agencies assess human exposure more accurately. This could involve assigning exposure values to study participants (in an epidemiological study) or calculating population exposure metrics for a neighborhood. Personal sensor data might directly feed into individual exposure profiles. Essentially, this step connects environmental data to the people who experience it.
- Health Impact Analysis: With improved exposure data, one can investigate health outcomes. This includes epidemiological studies (examining correlations between pollution levels and health metrics like asthma attacks, using the fine-grained exposure estimates from citizen data) and health impact assessments (estimating the disease burden or number of cases attributable to observed pollution levels). Citizen science data can thus inform quantitative evaluations of health risks.
- Risk Communication & Policy Translation: The findings are communicated back to the community and to decision-makers. This involves translating technical results into accessible messages (e.g., community meetings, reports, and apps). If the data reveals a significant health risk or injustice, this stage seeks to catalyze policy response, for instance, new regulations, targeted pollution controls, or community interventions. Importantly, citizens are part of this dialogue, advocating for their needs using the evidence they helped gather.
- Feedback Loops: Finally, the framework incorporates feedback loops. One loop is community feedback: seeing the data and outcomes often increases public awareness and can lead to behavior changes that reduce personal exposure (e.g., avoiding high-pollution areas at certain times). Another loop is policy feedback. If policies are implemented (e.g., traffic restrictions), the citizen network can monitor the effects, thereby closing the loop by evaluating policy impact on air quality and health. Positive results reinforce the value of citizen monitoring, and the cycle continues with perhaps new questions or expanded projects.

## Research Gaps and Future Directions

Despite considerable progress in the last decade, the integration of citizen science with air pollution and health research is still evolving. To shape the next generation of citizen science air quality research, it is important to understand the key research gaps and future directions, including technological innovations, methodological improvements, ethical considerations, and policy implications.

One major area for future work is enhancing the performance of low-cost sensors through technological innovation and advanced algorithms. Emerging sensor models are striving for better stability, lower detection limits, and the ability to measure additional pollutants (like NO_2_ or O_3_ with less drift). Research into new materials (e.g., nanomaterial-based sensing films) could yield more selective and sensitive low-cost detectors for gases [89]. In parallel, artificial intelligence (AI) and machine learning offer promising tools for calibration. Instead of simple linear correction factors, future citizen networks may deploy AI calibrators that learn from multi-sensor data in real time, adjusting for factors like humidity spikes, cross-pollutant interferences, or aging of sensors [90]. Some pilot studies (e.g., Bush et al., 2022) have shown that machine learning can significantly reduce error in field sensor data [91]. It is anticipated that “auto-calibration” algorithms embedded in sensor firmware or cloud platforms can continuously self-correct citizen data, making it more reliable with minimal human intervention [92]. Another frontier is sensor fusion via AI: combining inputs from a network of different sensors (PM, CO_2_, temperature, and others) to better infer pollutant levels and even source contributions [93]. These advancements could mitigate current limitations of low-cost sensors, expanding their applicability.

Methodologically, questions remain on the optimal design of citizen monitoring networks. How many sensors are needed, and where they should be deployed, to capture urban air pollution variability effectively? Studies like xAire began to tackle the question of the “minimal number” of sites for robust mapping [8], but further research using geostatistical techniques can help design sampling schemes that maximize information gain (perhaps using fewer sensors via smart placement). Mobile sensor networks (bikes, cars, even drones) raise questions about how to integrate moving data with static data, requiring new models for data fusion and interpolation. Developing algorithms that can assimilate crowd-sensed data of varying quality (indicative sensors, reference stations, satellite Aerosol Optical Depth (AOD)) into coherent high-resolution concentration fields is a ripe area for research [94]. Such data fusion, possibly leveraging techniques from meteorology (data assimilation) or computer science, could produce hybrid maps that take advantage of each data source’s strengths. Research can also explore near real-time modeling that ingests citizen data to forecast pollution (e.g., using citizens as a dense observational network feeding into air quality models for short-term forecasts) [95].

On the health side, a future direction is deeper integration of citizen science in epidemiological research [96]. Rather than treating citizen data only as input for researchers, future studies could involve citizens in tracking health outcomes alongside pollution. For example, a project could equip volunteers with asthma with personal air quality sensors alongside smart inhalers or structured health diaries, enabling direct correlation between individual exposure and health outcomes, such as wheezing episodes and inhaler use [97]. Scaling up such approaches requires careful attention to data privacy, the quality and governance of health data, and the maintenance of scientific rigour when relying on self-reported health measures. If successful, this approach can provide rich datasets linking exposure and effect at the individual level, helping unravel exposure-response relationships with fewer participants. Wearable technology advances (small sensors, biomonitors) and smartphones as data hubs will facilitate such studies [98]. A related frontier is using citizen networks to investigate acute health events, for instance, can real-time community air sensor data combined with syndromic health surveillance detect events like air-pollution related spikes in cardiac arrests or asthma emergency room visits? Integrating citizen environmental monitoring with public health monitoring systems could create an early warning system for extreme pollution health effects [99].

Future research should address how to sustain citizen science initiatives in the long run and how to scale successful models to new areas [100]. This has a social and organizational dimension: identifying incentives and support structures that keep volunteers engaged continuously. The role of schools, libraries, or community organizations as hubs for citizen science could be expanded. For instance, air monitoring can be incorporated into school STEM curricula to maintain youth involvement, while local libraries can loan out air quality sensors for community use. Policy support can also help institutionalize citizen networks, for instance, governments providing micro-grants or resources for community monitoring in each neighborhood. Another aspect is ensuring data interoperability and open access: as more projects emerge, standardizing protocols and data formats would allow separate citizen networks to connect into larger platforms (a sort of Internet-of-things for community air sensors) [101]. Research and development on robust, user-friendly data platforms (with visualization and analytics that non-experts can use) will be crucial so that citizen science data doesn’t languish unused, and so that communities can easily interpret their results [102].

With the growth of citizen science, ethical issues need more attention. One is data privacy: high-resolution pollution data can inadvertently reveal sensitive information (e.g., identifying pollution from a specific household’s activities). Projects must set guidelines on data sharing, anonymization of location if needed, and informed consent of participants about how data (and any personal information) will be used. Another issue is managing participant expectations and potential anxiety, when people see pollution data (especially if poor), it can cause stress or a feeling of powerlessness if not coupled with action pathways [103]. Ethical citizen science requires providing context and support: translating data to knowledge without unduly alarming, and empowering participants with options to reduce risk. There is also the potential burden on citizens: asking laypeople to take on work that agencies should possibly be doing. While citizen science can highlight problems, it might let authorities off the hook from expanding official monitoring networks. Ensuring that citizen efforts complement rather than replace governmental responsibility is an important consideration; ideally, citizen data should prompt agencies to improve their systems, not be seen as a substitute for free. Equity is another facet. Future projects must strive for inclusive participation. Research should explore best practices for recruiting and retaining participants from diverse socio-economic, racial, and age groups, perhaps learning from citizen science in other fields or from community organizing principles. Addressing language barriers, technological access, and providing clear benefits to participants (like personal exposure reports or community recognition) can help broaden involvement. An equitable approach also means acknowledging and crediting community contributions in publications and decisions, a cultural shift in how science values lay expertise [104].

Last, a frontier is how to formally integrate citizen science data into policy-making and regulatory processes [2]. Currently, regulatory bodies use citizen data informally (for supplemental information or community engagement). In the future, we may see structures where validated citizen network data are used in official air quality assessments or as part of permitting processes for local emission sources. For example, a city might adopt an ordinance that community sensor networks be consulted in evaluating air quality impacts of new developments, or an environmental agency might develop guidelines for how community data can trigger enforcement investigations. To enable this, there need to be standards and protocols, perhaps an ISO standard for “indicative air quality measurements by communities”, to ensure data quality and consistency at a level acceptable to regulators. Pilot collaborations between citizen scientists and regulators (e.g., the EPA’s Air Sensor Toolbox trials) will likely expand, generating the knowledge on how to blend grassroots and official monitoring effectively [105]. In policymaking, the hope is that citizen science leads to more responsive, transparent decisions. While some policy documents recognize the value of citizen science, there is a gap in actually using the results due to misalignment of timelines, data formats, or trust [106]. Bridging this gap is a key future task. It may involve developing clear communication channels (e.g., community data portals feeding into city dashboards), educating policymakers on interpreting citizen data, and co-developing policy questions with citizen input so that the data collected directly inform those questions [105].

Looking ahead, the coming decade offers significant opportunities to strengthen the role of citizen science in air-pollution and health research. By harnessing technological advances (including AI, wearables, and the IoT), improving approaches to community engagement and data integration, addressing ethical considerations, and building closer links with policy, citizen science can shift from the margins to a mainstream element of environmental health science. The vision is of participatory sensing networks that not only produce robust scientific data but also foster collective action and inform policymaking for cleaner air and healthier communities worldwide.

## Conclusion

This review underscores the transformative potential of citizen science in advancing air quality monitoring and environmental health research. By integrating participatory methods, low-cost sensing technologies, and co-created designs, citizen-led approaches enrich the spatial and temporal granularity of air pollution data while fostering public engagement and environmental justice. A consistent theme across literature is the ability of citizen-generated data to complement regulatory monitoring, offer hyperlocal insights, and catalyse community-informed interventions. However, critical gaps remain. Methodological heterogeneity, sensor drift, and limited calibration protocols hinder data comparability and scalability. Challenges persist in aligning citizen science outputs with epidemiological standards and policy frameworks. Furthermore, theoretical integration of citizen-generated evidence into exposure modelling and health-risk assessment remains underdeveloped. This review highlights the need for rigorous validation frameworks, hybrid sensor-network designs, inclusive engagement strategies, and longitudinal datasets to advance scientific and policy relevance. Future research should prioritise scalable calibration techniques, participatory epidemiology, and integration of AI-driven analytics to improve precision and uptake. Greater attention is also required to address ethical concerns, digital divides, and governance of citizen collected data. Ultimately, citizen science presents a vital frontier for democratising environmental data and co-producing actionable knowledge. By critically evaluating current evidence and outlining future priorities, this review provides a conceptual and practical foundation for integrating citizen science into air quality governance and public health research.

## Data Availability

No datasets were generated or analysed during the current study.
